# Inpainting with Separable Mask Update Convolution Network

**DOI:** 10.3390/s23156689

**Published:** 2023-07-26

**Authors:** Jun Gong, Senlin Luo, Wenxin Yu, Liang Nie

**Affiliations:** 1 Information System and Security & Countermeasures Experimental Center, Beijing Institute of Technology, Beijing 100081, China; 2 School of Computer Science and Technology, Southwest University of Science and Technology, Mianyang 621010, China

**Keywords:** image inpainting, image processing, separable mask update convolution, encoder-decoder network

## Abstract

Image inpainting is an active area of research in image processing that focuses on reconstructing damaged or missing parts of an image. The advent of deep learning has greatly advanced the field of image restoration in recent years. While there are many existing methods that can produce high-quality restoration results, they often struggle when dealing with images that have large missing areas, resulting in blurry and artifact-filled outcomes. This is primarily because of the presence of invalid information in the inpainting region, which interferes with the inpainting process. To tackle this challenge, the paper proposes a novel approach called separable mask update convolution. This technique automatically learns and updates the mask, which represents the missing area, to better control the influence of invalid information within the mask area on the restoration results. Furthermore, this convolution method reduces the number of network parameters and the size of the model. The paper also introduces a regional normalization technique that collaborates with separable mask update convolution layers for improved feature extraction, thereby enhancing the quality of the restored image. Experimental results demonstrate that the proposed method performs well in restoring images with large missing areas and outperforms state-of-the-art image inpainting methods significantly in terms of image quality.

## 1. Introduction

Image inpainting restores missing or damaged portions of an image, playing a crucial role in image coding and computational imaging. It fills in missing areas with plausible content, improving coding efficiency and fidelity. In computational imaging, it helps overcome challenges like occlusions and incomplete data, generating accurate scene representations. Overall, image inpainting facilitates efficient representation, transmission, and analysis of visual data, offering promising solutions for practical applications.

This technique has been widely applied in various fields, including medicine, military, and video processing, among others [1,2,3,4,5,6]. The early image inpainting methods were mainly based on traditional image processing techniques, such as texture synthesis, patch-based methods, and exemplar-based methods. Texture synthesis methods [7] typically fill in missing areas by replicating or generating textures from the surrounding image regions. Patch-based methods [8] use similar patches from the non-missing areas to fill in the missing regions. Exemplar-based methods [9], on the other hand, utilize a set of exemplar images to complete the missing regions by finding the most similar patches or structures from the exemplars. However, these early methods often suffer from a limited capability to handle complex structures and to generate realistic textures, resulting in visible artifacts and inconsistencies in the inpainted regions.

Recently, deep-learning-based approaches have emerged as the state-of-the-art for image inpainting, due to their ability to learn complex relationships and structures in the image data. These methods [10,11,12] typically involve using encoder-decoder networks to learn the context of the surrounding pixels, and then use this information to infer the missing content. Pathak et al. [13] were the first to apply convolutional neural networks to image restoration, and they designed a context encoder to capture the background information of images. Yang et al. [14] designed a dual-branch generator network, where one branch focuses on restoring the texture information of the image, while the other focuses on restoring the structural information, and then the results of the two branches are fused to improve the quality of the image restoration. Subsequently, due to the outstanding performance of generative adversarial networks in image restoration, many deep neural network architectures began to adopt adversarial learning strategies for image inpainting. For example, Yeh et al. [15] proposed an adversarial learning network consisting of a generator and discriminator, which can automatically generate high-quality restored images. The generator aims to generate complete images from the missing parts, while the discriminator is used to evaluate whether the generated results are similar to natural images. Iizuka et al. [16] proposed the concepts of the global discriminator and local discriminator. The global discriminator is used to detect the consistency of the overall image, while the local discriminator is used to detect the details and texture of local regions. The texture consistency of the restored results is ensured by evaluating the entire image and local regions. With the rapid development of deep learning, new technologies are constantly emerging in the field of image inpainting. For example, contextual attention mechanisms [17] can capture contextual information of different scales in the image, thereby improving the accuracy of image restoration. Partial convolution [18] only convolves known regions and ignores other missing parts, which can better handle missing areas. Gate convolution [19] can adaptively weight information from different positions to improve image restoration quality. Region normalization [20] can enhance the model’s generalization ability, thus making the image restoration results more accurate and robust.

However, it is challenging to model both the texture and structure of an image using a single shared framework. To effectively restore the structure and texture information of images, researchers, such as Guo et al. [21], have proposed a novel dual-stream network called CTSDG. This approach decomposes the image inpainting task into two subtasks, namely texture synthesis and structure reconstruction, further improving the performance of image restoration. This strategy allows for better handling of different feature requirements, resulting in enhanced quality and accuracy of the restored images.

Furthermore, existing image inpainting techniques typically provide only a single restoration result. However, image inpainting is inherently an uncertain task, and its output should not be limited. To address this issue, Liu et al. [22] introduced a new approach based on the PD-GAN algorithm. They considered that the closer the hole is to the center, the higher its diversity and strength. By leveraging this idea, they achieved satisfactory restoration results. This method introduces more diversity and realism in the restoration outcomes, enabling better adaptation to different inpainting requirements.

To address the restoration of boundary and high-texture regions, Wu et al. [23] proposed an end-to-end generative model method. They first used a local binary pattern (LBP) learning network based on the U-Net architecture to predict the structural information of the missing areas. Additionally, an upgraded spatial attention mechanism was introduced as a guide and incorporated into the image inpainting network. By applying these techniques, the algorithm aims to better restore the missing pixels in boundary and high-texture regions.

The aforementioned deep learning-based inpainting methods rely on the encoder-decoder to infer the context of small missing image areas. They then infer the texture details of the missing area based on the image features of the non-missing area and use local pixel correlation to restore the damaged image area. However, when the missing area of the image becomes larger and the distance between unknown and known pixels increases, these methods can produce semantic ambiguity due to the weakening of pixel correlation. Additionally, due to the limitations of convolution kernel size and a single convolution layer, the range of extracted information is too small to capture global structural information from distant pixels. As a result, it is challenging to repair larger missing areas with more semantics directly in one step.

Mou et al. [24] proposed a novel model called a deep generalized unfolded network (DGU-Net). This model integrates gradient estimation strategies into the steps of the gradient descent algorithm (PGD) to enhance the performance of the restoration process. However, it was not successful in handling large-area missing images. This indicates that there are indeed difficulties in effectively restoring images with extensive missing regions.

Inspired by the human learning process, by first learning some simple tasks and then gradually increasing the difficulty of the task, this learning strategy, from easy to difficult, can gradually learn a better performance model. The pixels inside the region are easier to repair. Therefore, Zhang et al. [25] proposed another progressive repair method, which progresses from the border of the missing region to the center. However, the progressive repair method must update the feature map in each iteration mapping back to the RGB space, resulting in a high computational cost. In response to this problem, Li et al. [26] designed the RFR-Net model to perform progressive restoration at the image feature level. That is, the input and output of the model need to be in the same space representation, which greatly saves computational costs. However, the RFR-Net model only uses the learnable convolution kernel to perceive the edge of the damaged area, ignoring the context information outside the receptive field. There are still some problems with blurred boundaries and incorrect semantic content that lead to repair results.

Aiming at the problem of huge amount of network parameters in image inpainting, we naturally think of optimizing the network structure and reducing unnecessary network layers. This paper uses the simplified encoder-decoder as the backbone of the generator. The end-to-end one-stage network dramatically reduces the complexity of the network compared to the progressive inpainting and multi-stage networks. Nevertheless, the cost of doing this is that the network may lose some ability to capture fine-grained texture details and global structural information, especially in large missing regions. In order to improve the restoration effect, this paper proposes a separable mask update convolution to reduce the interference caused by the missing regions in the image during the restoration process.

This paper presents three main contributions in the field of image inpainting:

• Lightweight end-to-end inpainting network: The paper introduces a novel lightweight end-to-end inpainting generative adversarial network. This network architecture, consisting of an encoder, decoder, and discriminator, addresses the complexity issue present in existing inpainting methods. It enables fast and efficient image restoration while maintaining high-quality inpainting results. The streamlined network design ensures computational efficiency and practicality;
• Separable mask update convolution: The paper proposes a unique method called separable mask update convolution. By improving the specific gating mechanism, it enables automatic learning and updating of the mask distribution. This technique effectively filters out the impact of invalid information during the restoration process, leading to improved image restoration quality. Additionally, the adoption of deep separable convolution reduces the number of required parameters, significantly reducing model complexity and computational resource demands. As a result, the inpainting process becomes more efficient and feasible;
• Superior inpainting performance: Experimental results demonstrate that the proposed inpainting network surpasses existing image inpainting methods in terms of both network parameters and inpainting quality. The innovative network architecture, coupled with the separable mask update convolution, achieves superior inpainting results with fewer parameters, reducing model complexity while maintaining high-quality restorations.

## 2. Related Work

### 2.1. Attention Mechanism

The attention mechanism can help the image inpainting model to find the most similar feature block from the non-missing area of the image according to the characteristics of the missing area, thereby improving the quality of image inpainting. Yu et al. [17] added an attention mechanism to the image inpainting network. The extracted feature information is divided into foreground and background areas, and the image feature blocks are matched in a long distance according to the similarity of the foreground and background. However, this image inpainting method ignores the correlation between the internal features of the missing area of the image. Therefore, Liu et al. [27] proposed a coherent semantic attention mechanism, which effectively improves the semantic consistency of the internal features in the missing area of the image. Since the features extracted by deep and shallow layers are not the same in convolutional neural networks, Zeng et al. [28] proposed a pyramidal context encoder network. The attention transfer network can transfer the attention information obtained from the high-level semantic features to the low-level features. This model is a restoration method that acts on the feature layer, which can improve the semantic consistency of the image after restoration. Literature [26] proposes a recurrent feature reasoning network, which works on the image feature level. In the process of feature reasoning, the designed knowledge consistent attention (KCA) module is added. The attention score determines the attention score of this module in the loop process, and the current attention score is jointly determined. This method can significantly save computational costs and achieve a more refined repair result. However, the features located in the missing area usually have a significant deviation, leading to the attention module’s wrong attention allocation. Finally, the model fills in incorrect texture details for some missing areas. Phutke et al. [29] applied wavelet query multi-head attention to image inpainting. Wavelet query multi-head attention is an attention mechanism that combines wavelet transforms with multi-head attention. This allows the model to attend to information from different representation subspaces at different positions, improving its ability to capture long-range dependencies and complex relationships between the input and output sequences.

### 2.2. Convolution Method

Convolution is a fundamental mathematical operation in deep neural networks to extract essential features from input signals or images. It has revolutionized computer vision and is widely used in various deep learning tasks, including image classification, object detection, segmentation, and image inpainting. Researchers commonly used valid convolution for feature extraction in the early stages of applying deep learning to image inpainting. During this period, they mainly focused on studying the restoration of regular square-shaped missing regions in the center of the image, as in the work of Pathak et al. [13] and Yu et al. [17]. Since the missing regions were regular, their impact on the restoration results was relatively low during the convolution kernel sliding process. However, what needs to be restored is often irregular regions. In this case, feature extraction using valid convolutions suffers interference from missing regions. Because the convolution kernel will cover many mixed windows of effective areas and invalid areas during the sliding process, this can lead to inaccurate learned features and thus affect image restoration results.

So, researchers began exploring using more advanced convolutional for image inpainting. The concept of partial convolution was first proposed by Liu et al. [18]. Partial convolution uses only valid pixels in the kernel to compute the output, ignoring invalid pixels (such as those in missing regions). This allows the convolution operation to focus on valid pixels, preventing missing regions from affecting the learned features. Partial convolution also has some limitations. One of the main limitations is that partial convolution is computationally expensive compared to regular convolution because it requires additional calculations to generate the mask. Additionally, partial convolution may not be suitable for cases where the missing regions occupy a large portion of the image because the valid pixels may not provide sufficient contextual information for restoration. Subsequently, in order to solve the problem that partial convolution cannot handle large areas of missing regions, gated convolution was proposed by Yu et al. [19]. Gated convolution is a variant of partial convolution that introduces an additional gating mechanism to control the flow of information through the convolutional kernel. The gating mechanism consists of a sigmoid function that generates a gating map to modulate the convolutional kernel’s feature responses. The gating map is used to selectively pass through the valid pixels in the convolutional kernel and suppress the invalid pixels in the missing regions. Liu et al. [30] used part of the convolution kernel to process the structure and texture features of the image to generate feature information with different scales. Due to the excellent performance of gated convolution in repairing irregularly missing regions, Ma et al. [31] proposed an improved version called dense gate convolution. This method incorporates the idea of dense connections, which allows information to flow freely within the network, thereby enhancing feature propagation and utilization.

Although the above convolution method solves the problem of large-area irregular mask image competition, there is still room for improvement in image restoration quality. Moreover, the problems of parameter expansion and increased calculation consumption caused by complex convolution methods have yet to be resolved but have intensified.

### 2.3. Progressive Image Inpainting

In view of the weak ability of convolutional neural networks in modeling long-distance pixel correlations between known long-distance regions (background regions) and regions to be inpainted (foreground regions), progressive image inpainting methods have been widely used in recent years. Xiong et al. [32] divided the whole inpainting task into three parts in sequence: perceiving the image foreground, completing object contours, and inpainting missing regions [33]. first predicted the structural information of the missing region of the image and then repaired the image according to the predicted structural information to improve the feature structure consistency between the repaired image and the real image. An excellent residual architecture in the full-resolution residual network proposed by Guo et al. [34] is helpful for feature integration and texture prediction. Furthermore, each residual block only reconstructs the specified missing regions to ensure image quality during the progressive inpainting process. Chen et al. [35] completed the image inpainting task step by step from the perspective of pyramid multi-resolution, during which low-resolution inpainting and high-resolution inpainting are performed in a cycle. Li et al. [36] stacked the visual structure reconstruction layer in the U-Net structure containing some convolutional layers. They reconstructed the structure and visual features of the missing area in a progressive manner. In this network, the updated structural information in each visual structure reconstruction layer is used to guide the filling of feature content to gradually reduce the missing area and finally complete the restoration task. Liao et al. [37] proposed a progressive image inpainting network that uses semantic segmentation information to constrain image content. However, these progressive image inpainting methods ignore the contextual information outside the receptive field of the convolution kernel. Shi et al. [38] proposed a multi-stage progressive inpainting method that divides the inpainting process into three stages: feature extraction, interactive inpainting, and reinforcement reconstruction. They used a dual-branch structure to focus on gradually restoring texture-level features. This approach avoids the redundant computation of previous cyclic progressive inpainting methods. Liu et al. [39] also used a dual-branch structure, but instead of having high-resolution and low-resolution branches, they focused on two progressive feature extraction branches for structure and texture feature extraction. This approach allows for the maximum restoration of the image’s structure and texture information.

### 2.4. GAN for Inpainting

Generative adversarial networks (GAN) is a deep learning model consisting of a generator and a discriminator. It has been widely used in image inpainting. The generator takes an image with missing regions as input and generates a repaired image, while the discriminator tries to distinguish between the generated repaired image and the real image. Through adversarial training, the generator gradually learns to generate realistic repaired images that visually resemble the real images. The application of GAN in image inpainting has the advantage of generating natural and preserving structural and texture features in the repaired results. In recent years, researchers have proposed different GAN-based image inpainting methods.

Guo et al. [21] proposed a novel two-stream network that models structure-constrained texture synthesis and texture-guided structure reconstruction in a coupled manner. The method, named "Conditional Texture and Structure Dual Generation (CTSDG)," incorporates a bi-directional gated feature fusion (Bi-GFF) module to exchange and combine structure and texture information, and a contextual feature aggregation (CFA) module to refine the generated contents using region affinity learning and multi-scale feature aggregation. Li et al. [40] introduced a novel multi-level interactive Siamese filtering (MISF) technique that combines image-level predictive filtering and semantic filtering on deep feature levels. Their method contains two branches: a kernel prediction branch (KPB) and a semantic and image filtering branch (SIFB). These branches interactively exchange information, with SIFB providing multi-level features for KPB and KPB predicting dynamic kernels for SIFB. This method leverages effective semantic and image-level filling for high-fidelity inpainting and enhances generalization across scenes. Chen et al. [3] proposed a feature fusion and two-step inpainting approach (FFTI). The method utilizes dynamic memory networks (DMN+) to fuse external and internal features of the incomplete image and generate an incomplete image optimization map. A generation countermeasure generative network with gradient penalty constraints is constructed to guide the rough repair of the optimized incomplete image and obtain a rough repair map. Finally, the coarse repair graph is optimized using the coherence of relevant features to obtain the final fine repair graph. Xia et al. [41] proposed an effective image inpainting method called a repair network and optimization network (RNON), which utilizes two mutually independent generative adversarial networks (GANs). The image repair network module focuses on repairing irregular missing areas using a generator based on a partial convolutional network, while the image optimization network module aims to solve local chromatic aberration using a generator based on deep residual networks. The synergy between these two network modules improves the visual effect and image quality of the inpainted images.

Although these methods have made significant progress in image texture and structure restoration, they also have certain limitations. The multi-stage, multi-branch, and multi-network nature of these methods leads to increased computational resource consumption and longer computation time. CTSDG, while advantageous in coupled texture synthesis and structure reconstruction, may have limitations when dealing with large-scale corruptions or missing regions spanning important areas of the image. The computational complexity of the Bi-GFF and CFA modules may restrict real-time performance in certain applications. MISF, which focuses on semantic filtering rather than fine-grained texture reconstruction, may experience a sharp performance decline when dealing with large missing areas requiring detailed texture recovery. The effectiveness of FFTI may depend on the quality and completeness of the external and internal features used for fusion, making it susceptible to interference from irrelevant information. RNON, which utilizes two independent GANs, requires more computational resources and time for training and faces an increased risk of mode collapse. The method’s repaired results also tend to exhibit over-smoothing.

## 3. Approach

Like a person completing a jigsaw puzzle alone, the image inpainting algorithm fills in the missing area by piecing together the surrounding pixels and keeping the contextual semantic information consistent with the image structure during the filling process. However, in the related work mentioned above, although progressive inpainting uses surrounding pixels to progressively restore missing pixels, they cannot maintain contextual image semantics and structural information well. At the same time, these algorithms have a large number of parameters and are computationally expensive. Therefore, this paper proposes a U-Net-like codec network with separable mask update convolution, significantly reducing network complexity.

### 3.1. Network Structure

**Figure 1:**
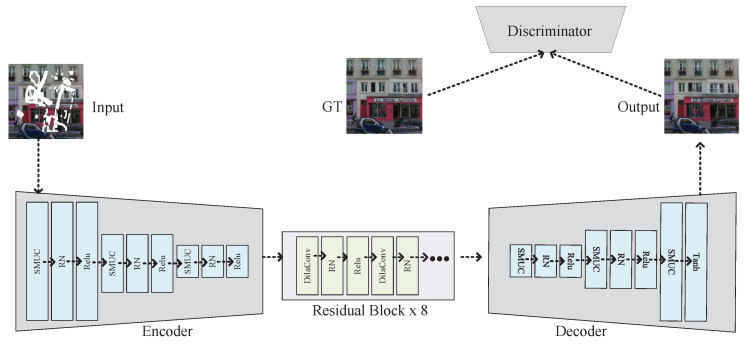
The structure of SMUC-net.

The proposed SMUC-net in this paper is a deep learning-based image restoration model whose backbone is a codec serving as the generator. Including components such as a discriminator, encoder, and decoder constructs an end-to-end learning framework that allows the entire restoration process to be completed within a unified framework. The overall structure of the SMUC-net is shown in Figure 1. Specifically, the encoder adopts separable mask update convolution modules and region normalization modules, which can effectively extract image feature information and optimize computation efficiency. Next, the image feature vector undergoes processing through eight residual blocks, which can effectively increase the depth and flexibility of the model while avoiding the problem of gradient vanishing. Finally, the decoder consists of separable mask update convolution layers and region normalization modules and uses the tanh function to activate the output result, obtaining the restored image.

In SMUC-net, the discriminator evaluates the similarity between the generated restored and original images, providing feedback mechanisms for model optimization. Among them, the loss function is the core of model optimization. This paper adopts multiple loss functions such as L1 loss, adversarial loss, perceptual loss, and style loss to train the model(the loss function is consistent with [20]). The L1 loss measures the pixel-level distance between the restored and original images, while the adversarial loss encourages the generator to produce more realistic restored images. The perceptual loss ensures the perceptual quality of the restored image by comparing the feature representations of the restored image and the original image. The style loss can make the restored image better conform to the style characteristics.

In recent years, deep learning has dramatically advanced the field of image restoration due to its remarkable performance in various applications. However, the challenges remain since the image restoration problem is inherently complex and challenging. SMUC-net represents a significant step forward in addressing these challenges, providing a powerful tool for restoring damaged or missing image information. Moreover, combining various loss functions and using a unified end-to-end learning framework in SMUC-net further enhance its effectiveness and versatility. Future research can explore improving the efficiency and accuracy of image restoration models like SMUC-net, extending their applicability to even more complex scenarios.

### 3.2. Separable Mask Update Convolution Modules

For irregular image inpainting tasks, missing regions may have arbitrary shapes and sizes, which means that traditional vanilla convolution-based inpainting algorithms are often incompetent. The following is the operation formula of vanilla convolution:

(1) $$\left(f*g\right)\left(x,y\right)=\sum_{i=-k}^{k}\sum_{j=-k}^{k}f\left(x-i,y-j\right)\cdot g\left(i,j\right)$$

Among them, *f* is the input image. *g* is the filter. *k* is the radius of the filter. (x,y) is the pixel position of the output image. The indices *i* and *j* represent the spatial location within the kernel matrix.

This formula says that each pixel value in the output image is the weighted sum of the filter at that location and surrounding pixels. It is not difficult to see from the formula that for the coordinate point position (x,y) of each channel of the input image *f*, the ordinary convolution will use the same shape of the filter *g* to perform convolution operations on it. This is because in ordinary convolution, the parameters of the filter are fixed independent of the pixel values in the input image. Therefore, no matter what the pixel values in the input image are, the same filter will be used for convolution. However, in tasks such as irregular image repair, since the missing area may have arbitrary shape and size, while the filter used by ordinary convolution is fixed, it is difficult to adapt to missing areas of different shapes and sizes, resulting in poor repair results.

**Figure 2:**
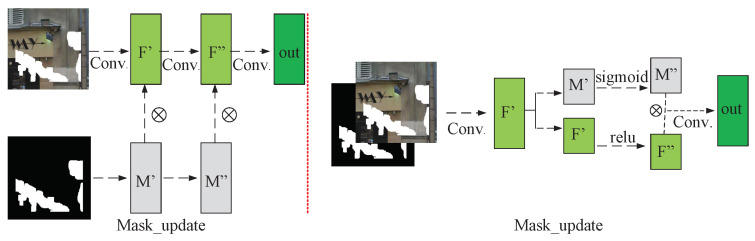
Illustration of partial convolution (**left**) and gate convolution (**right**).

In order to solve the above problems, it is necessary to control the interference of invalid information in the missing area of the image on the convolution result. Therefore, Liu et al. [18] proposed the concept of partial convolution. The calculation process of partial convolution needs a binary mask to assist. This mask consists only of 0 and 1. The mask position corresponding to the position where the pixel is 0 in the input image is also 0, and the corresponding mask position is 1 in other cases. Partial convolution uses a mask map to mark the areas of the input image that contain missing pixels. During convolution, only the valid pixel-containing areas are used for the convolution operation, while the invalid areas are excluded. This ensures that the missing areas do not interfere with the convolution result. Furthermore, a new mask image is generated during partial convolution, which guides the convolution operation of the subsequent layers. The operation flow of partial convolution is shown on the left side of Figure 2. Firstly, partial convolution is used to perform convolutional calculations on the input image, resulting in a new feature map. During this process, only the areas containing valid pixels are involved in the calculation, while the missing areas are excluded to avoid interfering with the convolution result. Then, the updated mask is used to perform a dot product with the feature map to obtain the input of the next layer in the network. Meanwhile, the updated mask is also used as the input for the next layer to guide the subsequent convolution operation. The partial convolution operation formula and mask update rules can be expressed as follows:

(2) $$x^{\prime }=\left\{\begin{array}{cc} \sum \sum w\cdot \left(x⊙\frac{1}{\mathrm{sum}(m)}\right), & \;\mathrm{if}\;\mathrm{sum}(m)>0\\ 0, & \;\mathrm{otherwise}\; \end{array}\right.$$

*w* represents the weight of the current convolution filter. *x* is the characteristic value of the current sliding window. ⊙ denotes element-wise multiplication. *m* represents the binary mask used for marking. (x,y) is the pixel position of the mask. After each partial convolution, mask-update follows the following strategy:

(3) $$\begin{array}{cc} \hat{m}\left(x,y\right) & =\left\{\begin{array}{cc} 1 & \;\mathrm{if}\;\mathrm{sum}(m)>0\\ 0 & \mathrm{otherwise} \end{array}\right. \end{array}$$

**Figure 3:**
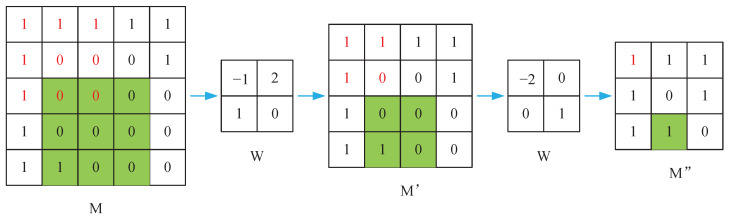
An illustration is provided to demonstrate the mask update mechanism employed by the partial convolution method.

The process of updating the mask is illustrated in Figure 3. When the mask region covered by the current convolutional window contains valid pixels, the corresponding region in the updated mask is marked as 1. However, the current masking method has a problem of considering all pixels as either valid or invalid, without considering the number of valid pixels. Therefore, in some cases, regions with only one valid pixel and regions with nine valid pixels are considered to have the same value. This is obviously unreasonable, especially as the network gets deeper, the actual information carried by regions of valid pixels can be very limited, as shown in the green region in the figure. The red area contains more effective pixels.

Based on the concept mentioned above, Yu et al. [19] proposed a novel approach to update the mask in the feature maps. They classified the feature maps into two groups based on the number of channels. They applied either sigmoid activation or relu activation on each group, as depicted on the right side of Figure 2. Specifically, the sigmoid activation operation was named GATE. It generated a weight map, also known as a `soft mask’, through which the pixels with lower weight values were deemed to have a higher probability of containing invalid information. The soft mask was then multiplied with the activated feature map to reduce the confidence of invalid pixels in the feature map, thereby allowing the network to focus on the most informative regions. This process improved the quality of feature representations and helped eliminate the negative influence of noise and irrelevant information.

The gate convolution proposed by Yu et al. [19] is undoubtedly effective in improving the quality of feature representations. However, this approach also presents two notable challenges that need to be addressed. Firstly, the feature maps are split into two groups, which require twice as many convolution kernels and double the number of parameters in the model. This operation increases the computational cost and storage requirements, making deploying the model on resource-limited devices challenging. Secondly, the equal division of feature maps may reduce the feature space available for learning and limit the expressive power of the model.

This paper proposed a novel approach called separable mask update convolution to overcome these limitations. This approach addresses the challenges mentioned above by introducing a two-step process separating the convolutional and gating operations. Specifically, the separable mask update convolution first applies a regular convolutional operation to the input feature map, generating a set of intermediate feature maps. Then, a gating operation is performed on the intermediate feature maps to obtain a set of weight maps. By separating the two operations, the separable mask update convolution can reduce the number of convolution kernels and parameters in the model while achieving similar or even better performance than the original gate convolution approach. Moreover, the separable mask update convolution approach allows more flexibility in designing the model architecture and improves the model’s ability to learn complex representations.

**Figure 4:**
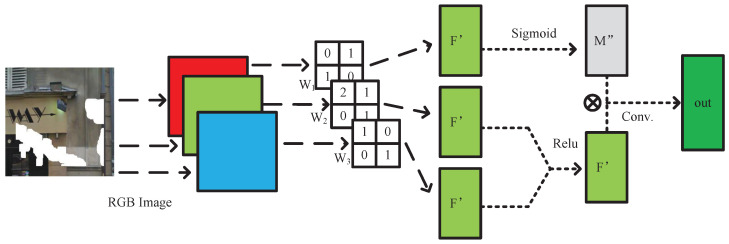
Illustration of our separable mask update convolution.

Based on the operation principle shown in Figure 4, the separable mask update convolution method follows a few steps. Firstly, the group parameter of the convolution kernels is set to be the same as the number of input channels, which results in an equal number of output feature maps as the input channels. Let us assume the number of input channels is $N_{c}$. Therefore, $N_{c}$ feature maps are generated.

Next, the $N_{c}$ feature maps are divided into two groups with a proportion of $N_{c}-1:1$. The relu function activates the first group. In contrast, the sigmoid function activates the second group. The reason for using two different activation functions is to provide diverse nonlinear transformations to the feature maps. After activation, the two groups are multiplied to obtain the weighted feature maps.

Finally, the weighted feature maps are passed through the convolutional layer, which consists of filters with a kernel size equal to 1. This convolutional layer helps to expand the output channels and generates a new set of feature maps with increased depth. By using the separable mask update convolution method, the model can learn more complex representations with fewer parameters, resulting in better performance and faster convergence during training.

The convolution method proposed in this paper can significantly reduce the number of parameters compared to gate convolution. It also optimizes the number of feature maps required for mask update and improves the information utilization rate. Below are the formulas for calculating parameters in gated convolution and separable mask update convolution.

The calculation of parameters for gate convolution [19] can be expressed as:

(4) $$\begin{array}{cc} N_{gc} & =K_{size}\times K_{size}\times N_{out}\times N_{in}\\ \; & =\left(K_{size}\times K_{size}\times N_{in}\right)\times N_{out} \end{array}$$

The calculation of parameters for separable mask update convolution can be expressed as:

(5) $$\begin{array}{cc} N_{smuc}\; & =K_{size}\times K_{size}\times N_{in}+N_{out}\times 1\times 1\times N_{in}\\ \; & =\left(K_{size}\times K_{size}+N_{out}\right)\times N_{in}\\ \; & =\left(K_{size}\times K_{size}\times N_{in}\right)\times \left(1+\frac{N_{out}}{K_{size}\times K_{size}}\right) \end{array}$$

The formulas show that the number of parameters required for the two convolution methods, gate convolution (GC) and separable mask update convolution (SMUC). The variables $N_{gc}$ and $N_{smuc}$ respectively represent the number of parameters required for each method. The kernel size is denoted by $K_{size}$, while $N_{in}$ and $N_{out}$ represent the number of input and output channels. It can be observed that when $K_{size}=1$, SMUC has more parameters than GC due to the presence of an additional $N_{in}$ term. However, for larger kernel sizes, SMUC requires fewer parameters than GC.

### 3.3. Region Normalization

The separable mask update convolution effectively reduces the impact of invalid information in the missing region on the restoration results during the convolution process. In the normalization layer, regular normalization methods cannot completely avoid the interference of invalid information in the missing region on the restoration results, especially when the missing region is large. Traditional normalization methods usually normalize the pixel values of each feature map to reduce the covariance between feature maps, thereby enhancing the robustness and generalization ability of the network. However, for the missing region, as the pixel values in the missing region are usually zero or very small, such normalization methods often cannot effectively reduce the invalid information in the missing region but may increase the impact of noise, further affecting the quality of the restoration results. To solve this problem, some unique normalization methods for the missing region have emerged in recent years, such as the normalization method based on local variance and the normalization method based on masks. These methods can better remove invalid information in the missing region through special processing of the missing region, thereby improving the quality of the restoration results.

**Figure 5:**
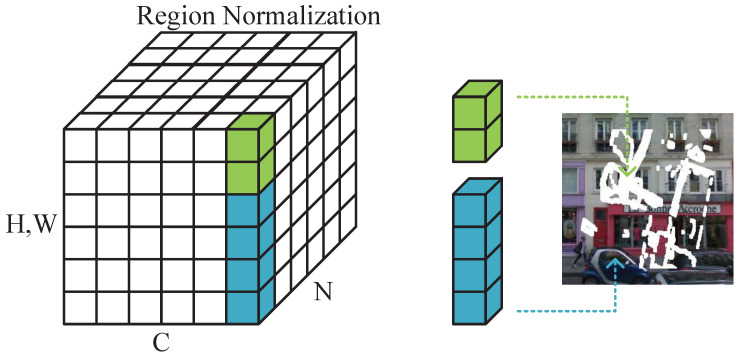
This figure demonstrates the proposed Region Normalization (RN) method, which applies different normalization parameters to pixels of different regions. Specifically, pixels within the same region, represented by green or blue color, share the same mean and variance during normalization. Notably, the corrupted and uncorrupted regions of the input image are normalized by different mean and variance parameters.

In this paper, a novel technique called Region Normalization (RN) [20] is introduced to address the challenge of mean and variance shifts in the normalization process. The method is specifically designed for use in the early layers of the inpainting network, where the input feature contains large corrupted regions that result in significant mean and variance shifts. RN addresses this issue by separately normalizing and transforming the corrupted and uncorrupted regions, thus preventing information mixing. In specific operations, the region normalization method divides each input feature map according to its four dimensions (N, C, H, W). Then it divides it into damaged and undamaged regions based on whether there is damaged data in each region. As shown in Figure 5, the height and width of each feature map in the batch can be divided into multiple block regions. The green area represents the damaged data, and the blue area represents the undamaged data, which are normalized separately.

### 3.4. Loss Function

When performing image restoration, the loss function is a crucial part. By defining an appropriate loss function, we can guide the model to learn how to better restore the image. In this paper, various loss functions are adopted for image inpainting, including perceptual loss, reconstruction loss, adversarial loss and style loss. These four loss functions correspond to the reconstruction error, feature similarity, adversariality, and style similarity between the inpainted image and the real image, respectively. The total loss of the generator can be expressed as a weighted sum of these four loss functions, where each loss function has its own weight. The following are the specific formulas of reconstruction loss, perceptual loss, confrontation loss and style loss:

(6) $$L_{recon}={∥\hat{y}-y∥}_{2}^{2}$$

(7) $$L_{perceptual}=\frac{1}{N}\sum_{i=1}^{N}{∥\phi_{i}\left(\hat{y}\right)-\phi_{i}\left(y\right)∥}_{2}^{2}$$

(8) $$L_{adv}\left(G,D\right)=\log D\left(y\right)+\log\left(1-D\left(G\left(z\right)\right)\right)$$

(9) $$L_{style}=\frac{1}{N}\sum_{i=1}^{N}{∥\mathrm{Gram}\left(\phi_{i}\left(\hat{y}\right)\right)-\mathrm{Gram}\left(\phi_{i}\left(y\right)\right)∥}_{2}^{2}$$

Among them, $\hat{y}$ is the image generated by the generator, *y* is the real image, $\phi_{i}$ represents the feature representation of the *i*-th layer in the pre-training model, specifically pool1, pool2, and pool3 layers. Therefore, in this paper, L=3, $N_{i}$ represents the number of elements in $\phi_{i}$, *D* is the discriminator, *G* is the generator, *z* is the input image, and Gram represents the Gram matrix. The total loss is then expressed as:

(10) $$L_{adv}=\frac{1}{N}\sum_{i=1}^{N}\log\left(D\left(\hat{y}\right)\right)$$

The weight coefficients α,β,γ,δ are used to control the contribution of each loss function to the overall loss. The total loss function includes four losses, which constrain the generator from different perspectives and effectively improve the quality and effectiveness of image restoration. In this paper, α,β,γ, and δ are set to 1, 0.1, 0.1, and 250, respectively.

## 4. Experiment

### 4.1. Experiment Setup

For our experiments, we chose to use three common datasets for image inpainting, namely Paris Street View [13], Celeba-HQ [42] and Places2 [43]. The Paris Street View dataset, proposed by researchers from ParisTech and INRIA (French National Institute for Research in Computer Science and Automation), consists of 15,000 high-resolution images capturing street views and buildings in Paris. This dataset is commonly used in research on scene understanding, image inpainting, and image synthesis in computer vision and image processing tasks.

The Celeba-HQ dataset, introduced by Ziwei Liu et al. in 2018, is an extension of the Celeba (Celebrities Attributes) dataset with higher quality images. It contains 30,000 high-quality images of celebrity faces that have been carefully curated and processed. The Celeba-HQ dataset is widely used for training and evaluating computer vision algorithms related to face recognition, face generation, and face inpainting tasks.

The Places2 dataset is a widely used large-scale image dataset proposed by researchers from the International Computer Science Institute (ICSI) and the Berkeley Vision and Learning Center (BVLC). It comprises over one million carefully curated images, capturing diverse real-world scenes such as indoor and outdoor environments, natural landscapes, urban street views, and office spaces. These high-quality images, with resolutions of 256 × 256 or 512 × 512 pixels, exhibit rich semantic and visual diversity, covering various scene types, lighting conditions, and perspectives. The Places2 dataset serves as a vital benchmark for scene understanding, image generation, image classification, and other computer vision tasks, enabling researchers to train and evaluate their models and driving advancements in the field.

In the study, the first 14,900 images from the Paris Street View dataset were used for training the model, while 100 images were reserved for testing. As for the Celeba-HQ dataset, the first 28,000 images were used for training, and the remaining 2000 images were used for testing. This data split ensures that the model is exposed to representative image samples during both training and testing, enabling accurate assessment of the performance and generalization capability of the image inpainting algorithm across different datasets. For the Places2 dataset, we followed the official partitioning of training and testing sets. We created our own training set by selecting the first 100,000 images from the complete Places2 training set. Similarly, our testing set was formed by selecting the first 2000 images from the complete Places2 testing set.

To create the masks for our experiments, we used the irregular mask dataset [18], which consists of a variety of randomly generated masks with different shapes and sizes. Liu et al. created and released the dataset of irregular masks when they proposed partial convolution. It has become one of the most widely used public datasets for irregular mask image inpainting among existing image restoration methods. We divided the masks into five categories based on the proportion of missing area, namely 10–20%, 20–30%, 30–40%, 40–50%, and 50–60%. Each category contained 2000 masks.

To train our model, we used a single NVIDIA GeForce GTX 1080Ti graphics card and set the number of epochs to 10. We continued training until the model converged and achieved satisfactory results on our test dataset.

### 4.2. Quantitative Comparison

In addition to describing the proposed method, this paper also compared it with other commonly used image restoration methods, including Region Normalization (RN) [20], Conditional Texture and Structure Dual Generation (CTSDG) [21], and Multi-level Interactive Siamese Filtering (MISF) [40]. These comparison methods have shown good performance in recent years in the field of image restoration. The comparison was carried out on two datasets, Celeba-HQ and Paris Street View, and the test results were presented in Table 1 and Table 2, respectively. The two metrics used to evaluate the performance were PSNR and SSIM, which reflect the pixel similarity and structural similarity between the inpainting results and the original image, respectively.

Finally, we compared the multi-level interactive Siamese filtering (MISF) [40], repair network and optimization network (RNON) [41], and features fusion and two-steps inpainting (FFTI) [3] methods on the comprehensive Places2 dataset. In this comparative experiment, we excluded the experiments on the extreme conditions of extremely small and extremely large missing areas. The experimental results were focused on the common range of 20% to 50% missing regions. The experimental results are presented in Table 3.

The results show that the proposed method outperformed the comparison methods in terms of PSNR and SSIM on both datasets, especially in the 10% to 60% range of missing area. For example, on Celeba-HQ, the proposed method achieved a PSNR improvement of 1.06–1.6 dB and an SSIM improvement of 0.026–0.127, depending on the scale of the missing area. On Paris Street View, the PSNR improvement was 0.827–1.69 dB and the SSIM improvement was 0.019–0.137. These results indicate that the proposed method can recover more structural information, especially when repairing large missing areas.

On the comprehensive Places2 dataset, our proposed method continues to exhibit a distinct advantage over recent state-of-the-art approaches in the restoration of large-scale missing image regions. Particularly for missing areas exceeding 30%, as the extent of the missing region increases, our method consistently outperforms other techniques in terms of both PSNR and SSIM metrics. Furthermore, in the restoration of small-scale missing regions (below 30%), although our method may not surpass the performance of the highly effective FFIT method, the discrepancy in PSNR values between the two approaches remains minimal.

Furthermore, the proposed method has fewer parameters compared to the RN method, which is a large-scale network with the fewest parameters. Additionally, when compared to the RNON method, our method only has one-third of the parameter count. In comparison to the FFIF method, the proposed approach reduces the number of parameters by nearly two-thirds. Hence, the proposed method not only demonstrates better performance but also exhibits a more compact structure.

### 4.3. Qualitative Comparison

**Figure 6:**
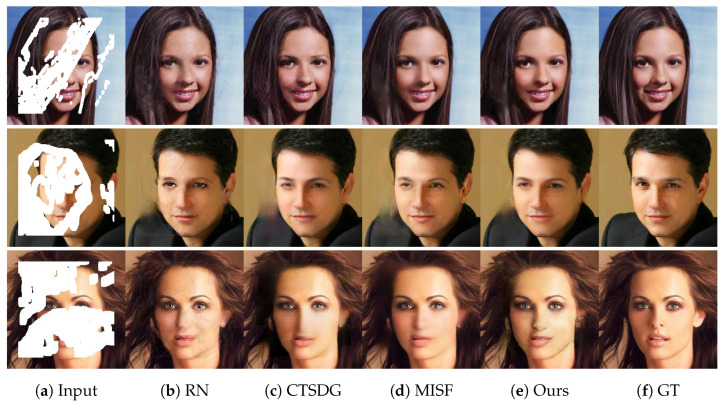
The result display of our method on CelebA-HQ dataset under different missing data scales.

**Figure 7:**
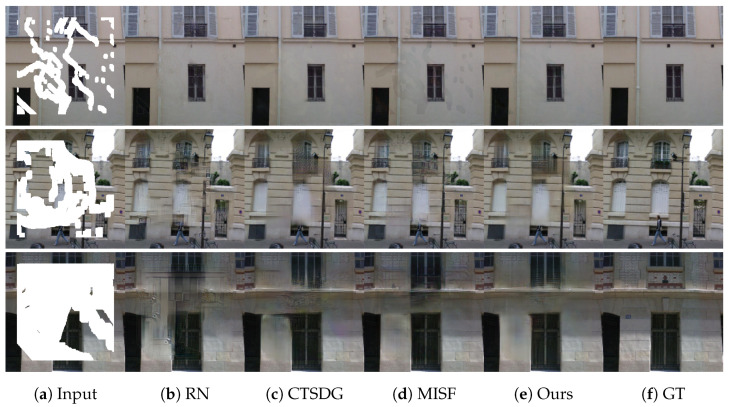
This is the result display of our method on Paris street view dataset under different missing data scales.

Figure 6 and Figure 7 show the restoration results of two datasets under different missing ratios. The results demonstrate that our proposed method outperforms other methods in restoration effectiveness at any scale. It is worth noting that the performance of the existing methods and our proposed method vary when dealing with different types of images. For example, on the CelebA-HQ dataset, which consists of facial images, the SMUC-net produces more natural-looking results with softer facial contours. In contrast, the other methods tend to generate slightly more artificial-looking images. This indicates that our proposed method is more effective in preserving the natural features of facial images.

In contrast, on the Paris Street View dataset, which includes a variety of urban scenes, our method performs better in restoring texture details of objects such as branches and windows. This is due to our method’s ability to recover the structural information of the missing areas, which is crucial in restoring the texture details of objects.

Another significant advantage of our method is its ability to handle large missing areas. As demonstrated in Figure 7, our method can effectively recover the structural information of the missing areas and generate more realistic images than other methods, even when up to 60% of the image is missing.

**Figure 8:**
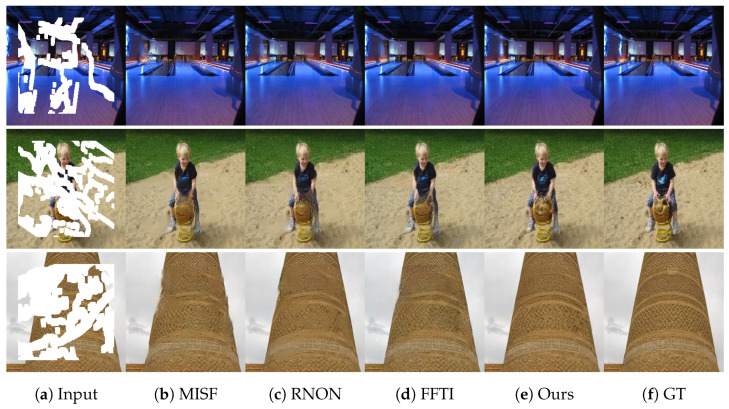
This is the result display of our method on Places2 dataset under different missing data scales.

As shown in Figure 8, on the Places2 dataset, it is easy to observe that our proposed restoration method outperforms other methods in terms of preserving more detailed information in large-scale irregular missing regions. Notably, it effectively restores finer details such as the texture on buildings or the intricate details of a child riding a toy horse.

However, it should be noted that our method may still encounter challenges in restoring certain types of objects, such as text or other highly structured elements. In such cases, our method may be unable to repair them successfully. Nevertheless, our method produces fewer artifacts and more realistic results compared to other methods.

In summary, our proposed method outperforms existing methods in restoring natural features and texture details, handling large missing areas, and producing more realistic results with fewer artifacts. These findings demonstrate the potential of our method for various applications, such as image editing and restoration.

### 4.4. Ablation Study

To validate the effectiveness of the proposed method in this paper, we conducted ablation experiments on the CelebA dataset. The mask used in this case is based on a general damage range of 30% to 40%. Following the proposed repair method, we restored separable mask update convolutions to ordinary convolutions and normalized the region normalization layer to a standard normalization layer as the base model. Then, we separately added separable mask update convolutions and region normalization layer for training and finally added both methods to the base model for training. The final model we obtained is the proposed repair method in this paper. The experimental results are shown in Table 4.

After conducting experimental comparisons, we see that both replacing separable mask update convolution and region normalization layers can significantly improve the restoration performance compared to the original basic network architecture. This indicates that both methods are effective in improving restoration performance. Because both separable mask update convolutions and region normalization can reduce the interference of invalid pixels in the damaged area to some extent, they contribute to improving the repair results. It is worth noting that the core separable mask update convolution module proposed in this paper also plays an essential role in reducing network parameters. Ultimately, our experimental results show that the proposed SMUC-net achieves the best results in both restoration performance and network parameter count.

## 5. Conclusions

This article proposes a simple encoder-decoder network that combines separable mask update convolutions and region normalization techniques to improve image restoration. The network parameters are significantly reduced using separable mask update convolutions instead of traditional convolution operations. Additionally, the separable mask update mechanism can preserve more feature information and reduce the impact of invalid pixels by providing different weights to masked and unmasked areas, further enhancing the restoration effect.

Furthermore, the article introduces the region normalization technique to provide different means and variances for masked and unmasked areas. This method can reduce the influence of masked areas on the restoration results, thereby improving the accuracy of image restoration. Through experimental comparisons, we found that the proposed method achieved a good restoration effect and network parameter quantity results.

Experimental results on the Celeba-HQ and Paris Street View datasets show that our proposed method outperforms FFTI by 1.06–1.6 dB and 0.827–1.69 dB in terms of PSNR and by 2.6% to 12.7% and 1.9% to 13.7% in terms of SSIM under damage rates of 10% to 60%. Moreover, our method successfully reduces the parameter quantity by 16.58 M, making it the model with the minor parameters but the best restoration results.

The image inpainting method proposed in this paper has achieved significant improvements in terms of network parameters and inpainting quality. However, the main limitation of our approach is it lacks interactivity. A possible future direction could be to incorporate user guidance information into the inpainting process, which may provide more opportunities for user participation and customization. In addition, robot painting [44,45] is also a promising application direction. In practical applications, our image inpainting method can assist robots in better filling in missing image content.

## Figures and Tables

**Table 1:** This is a demonstration of the quantitative results of the method in this paper at different defect scales on the CelebA-HQ dataset (M = $2^{20}$).

	Mask	RN [20]	CTSDG [21]	MISF [40]	Ours
PSNR↑	10–20% 20–30% 30–40% 40–50% 50–60%	29.339 26.344 24.060 22.072 20.274	29.842 26.550 24.652 23.122 20.459	29.868 27.154 24.993 23.185 20.455	**31.472** **28.321** **26.053** **24.420** **21.578**
SSIM↑	10–20% 20–30% 30–40% 40–50% 50–60%	0.919 0.866 0.811 0.749 0.667	0.935 0.878 0.832 0.778 0.686	0.933 0.889 0.838 0.780 0.687	**0.959** **0.926** **0.889** **0.876** **0.814**
Parameters↓		25.34 M	31.26 M	27.60 M	**11.02 M**

**Table 2:** This is a demonstration of the quantitative results of the method in this paper at different defect scales on the Paris street view dataset (M = $2^{20}$).

	Mask	RN [20]	CTSDG [21]	MISF [40]	Ours
PSNR↑	10–20% 20–30% 30–40% 40–50% 50–60%	29.237 26.678 24.517 22.556 20.424	30.375 27.188 25.424 23.412 20.844	30.042 27.465 26.059 24.057 21.416	**31.732** **28.680** **26.934** **25.192** **22.243**
SSIM↑	10–20% 20–30% 30–40% 40–50% 50–60%	0.912 0.848 0.781 0.707 0.598	0.930 0.875 0.819 0.743 0.647	0.926 0.877 0.833 0.761 0.655	**0.945** **0.928** **0.873** **0.832** **0.792**
Parameters↓		25.34 M	31.26 M	27.60 M	**11.02 M**

**Table 3:** This is a demonstration of the quantitative results of the method in this paper at different defect scales on the Places2 dataset (M = $2^{20}$).

	Mask	MISF [40]	RNON [41]	FFTI [3]	Ours
PSNR↑	20–30% 30–40% 40–50%	26.115 24.260 22.140	26.742 25.944 23.386	**27.657** 26.387 23.671	27.510 **26.957** **24.327**
SSIM↑	20–30% 30–40% 40–50%	0.863 0.795 0.741	0.894 0.846 0.798	**0.909** 0.862 0.811	0.896 **0.883** **0.872**
Parameters↓		27.60 M	31.46 M	34.20 M	**11.02 M**

**Table 4:** This table provides a detailed display of the inpainting results of different network models. The base model does not include the separable mask update convolution and region normalization methods, while the SMUC-net includes both mechanisms (M = $2^{20}$).

Models	PSNR	SSIM	Parameters
Base Model	23.76	0.799	12.83 M
Base Model+SGC	25.67	0.855	**11.02 M**
Base Model+RN	24.94	0.832	12.83 M
SMUC-net	**25.92**	**0.871**	**11.02 M**

## Data Availability

Not applicable.

## Abbreviations

The following abbreviations are used in this manuscript:

SMUC	Separable Mask Update Convolution
GC	Gate Convolution
RN	Region Normalization
CTSDG	Conditional Texture and Structure Dual Generation
MISF	Multi-level Interactive Siamese Filtering
RNON	Repair Network and Optimization Network
FFTI	Features Fusion and Two-steps Inpainting
